# Genetic and Environmental Factors in Complex Neurodevelopmental Disorders

**DOI:** 10.2174/138920207783591717

**Published:** 2007-11

**Authors:** K.M.J van Loo, G.J.M Martens

**Affiliations:** Department of Molecular Animal Physiology, Donders Institute for Neuroscience, Nijmegen Center for Molecular Life Sciences (NCMLS), Faculty of Science, Radboud University Nijmegen, Geert Grooteplein Zuid 28, 6525 GA Nijmegen, The Netherlands

**Keywords:** Neurodevelopmental disorders, susceptibility genes, environmental factors, gene-environment interactions, association studies, linkage analysis.

## Abstract

Complex neurodevelopmental disorders, such as schizophrenia, autism, attention deficit (hyperactivity) disorder, (manic) depressive illness and addiction, are thought to result from an interaction between genetic and environmental factors. Association studies on candidate genes and genome-wide linkage analyses have identified many susceptibility chromosomal regions and genes, but considerable efforts to replicate association have been surprisingly often disappointing. Here, we summarize the current knowledge of the genetic contribution to complex neurodevelopmental disorders, focusing on the findings from association and linkage studies. Furthermore, the contribution of the interaction of the genetic with environmental and epigenetic factors to the aetiology of complex neurodevelopmental disorders as well as suggestions for future research are discussed.

## INTRODUCTION

The term neurodevelopmental disorder is a relatively new term and includes a group of disorders with severely affected behavioral features caused by alterations in early brain development. Most neurodevelopmental disorders are associated with a life-long endurance and have a severe impact on normal brain functioning, leading to affected behavior often resulting in large economical, emotional and physical problems, not only for the individual but also for the family and society as a whole. The various neurodevelopmental disorders show similar features, including brain dysfunctioning (such as difficulties in sensor and motor systems, problems with speech and language) and a number of cognitive impairments (e.g. in learning and organizational skills)*.* Schizophrenia, autistic disorders, attention deficit hyperactivity disorder (ADHD), bipolar disorder, mental retardation and Tourette’s syndrome are some of the more common neurodevelopmental disorders, but also Rett syndrome, immunodeficiency, centromeric region instability, facial anomalies (ICF) syndrome and X-linked alpha thalassemia/mental retardation (ATR-X) syndrome are considered neurodevelopmental disorders (Table **1**).

## NEURODEVELOPMENTAL DISORDERS AND GENETIC AETIOLOGY 

Neurodevelopmental disorders can be divided into four subgroups, based on their (mostly hypothetical) genetic aetiology (Table **1**). The first subgroup is characterized by aneuploidy (an abnormal number of chromosomes). The most well-known neurodevelopmental aneuploidy is Down’s syndrome with a trisomy of chromosome 21*.* Disorders of the second subgroup contain chromosomal micro-deletions, such as the deletion of chromosomal region 7q11.2 (which harbours more than 20 genes) in William’s-Beuren syndrome. In each neurodevelopmental disorder of the third subgroup, only a single gene is affected. For example, the fragile X syndrome is a genetic disorder caused by a mutation (CGG repeat expansion) of the fragile X mental retardation 1 (FMR1) gene on the X chromosome. The neurodevelopmental disorders with a complex aetiology, such as autism and schizophrenia, comprise the fourth subgroup and are thought to be caused by (a combination of) genetic, environmental and epigenetic factors. In this review, we focus on the neurodevelopmental disorders with a complex aetiology and the current thoughts on their genetic, environmental and epigenetic aetiologies. 

## IDENTIFICATION OF SUSCEPTIBILITY LOCI AND GENES 

Twin, family and adoption studies have revealed an unambiguous role for genetic factors in the aetiology of complex neurodevelopmental disorders that can even exceed an estimated heritability of 90% (in autism; Table **2**). Although a genetic component is thus clearly involved in the aetiology of a complex neurodevelopmental disorder, it is still elusive which gene (or genes) is responsible for its pathogenesis. Historically, the dopamine and also the glutamate neurotransmission system have often been implicated to play a role in neurodevelopmental pathogenesis. However, since many recently identified susceptibility genes have been found not to be related to either of the two neurotransmitter systems, restriction to these systems is no longer justified. To identify susceptibility genes and to better understand the pathophysiology of complex neurodevelopmental disorders, many studies utilizing genetic, biochemical, pharmacological, neurological and cognitive neuroscience techniques have been performed. In this section, we summarize the genetic approaches that have been used to identify risk factors at specific loci and genes.

### Linkage Studies

Linkage analysis is a method to locate disease-related loci using DNA markers across the genome that travel with a disease within families. The main advantage of linkage analysis is that it involves family-based analysis, and thus eliminates the problem of ethnical stratification. However, linkage analysis has a relatively low power to detect small-effect variations [1].

### Association Studies

Numerous association studies have been performed to test for association between genetic variations and neurodevelopmental disorders [2, 3]. Compared to linkage analysis, one important advantage of association studies concerns its improved power when equal cohort sizes are used [4]. In association studies, the genotype or allele frequencies of genetic variations between patients and controls (non-related individuals; case-control design) or between parents and their offspring (related individuals; family-based design) is compared. For the case-control design, a more than by chance predicted difference in the frequency of a single-nucleotide polymorphism (SNP) between the cases and controls indicates that the specific polymorphism may increase or decrease risk for the disorder, or is in linkage disequilibrium with a nearby genetic variant. The frequencies of genetic varations may vary among individuals from a different geographical or ethnical background and therefore a well-defined cohort is necessary. For family-based association studies, the parents function as the controls for the affected offspring (so-called trio-study). If the SNP is transmitted from the parents to the offspring as expected by chance alone, no association with the disorder is present. Transmission of the SNP at a higher degree than expected by chance suggests association of the genetic marker with the affected phenotype. 

Having decided on the study design and study samples, the next step is to select appropriate candidate genes. In general, a gene is selected with some *a priori* relationship with the disorder, based on its localization (i.e. the gene is located in a chromosomal region with a significant linkage), or proposed function in the pathogenesis of the disorder (e.g. the gene belongs to the dopamine or serotonin pathway in association studies for schizophrenia pathogenesis). Genome-wide association (GWA) studies can now also be performed, whereby large numbers of DNA polymorphisms are analyzed in one experiment. 

### Copy Number Variations

Recently, it became clear that besides mutations and SNPs (both coding and non-coding alterations) also genomic rearrangements and gene-dosage imbalances (duplications, deletions and inversions) play a role in the pathogenesis of a number of nervous system disorders [reviewed in 5]. These structural variants are common and ubiquitous in the genome and can range from kilobases to megabases in size. The human genome contains at least 1447 copy-number variants (CNVs), covering 360 megabases and comprising 12% of the genome [6]. Previous knowledge of CNVs in relation to diseases was limited due to insufficient methods to detect CNVs. Only large CNVs detected with cytogenetic techniques, such as G-banding (Giemsa staining) and fluorescence in situ hybridization, have been previously identified. The advent of high-resolution genome-wide methods has significantly improved the power to detect CNVs. 

At present, one of the most attractive techniques to detect CNVs is comparative genome hybridization (CGH) using DNA microarrays containing genomic DNA probes (e.g. bacterial artificial chromosome clones, cDNA clones or oligonucleotides). The CGH technology allows a genome-wide screening with a relatively high resolution (with the resolution depending on the number, distribution and lengths of the probes present on the array), and may be particularly useful for the identification of CNVs that are too small to detect *via *routine cytogenetic analyses. Another type of array that can be used for detecting CNVs is the genome-wide SNP array. Besides normal SNP analysis (i.e. the identification of a single-base polymorphism), these arrays also give intensity information and thereby the corresponding copy number of a genomic region. Once CNVs have been detected, studies for locus-specific CNV association have to be designed, such as targeted quantitative and semiquantitative PCR, multiplex ligation-dependent probe amplification or dynamic allele-specific hybridization.

### mRNA and Protein Expression Profiling 

The principle of mRNA and protein profiling is to identify genes that are differentially expressed in selected tissues of cases and (matched) controls. One of the promising strategies is the use of human post-mortem brain tissues, from which nowadays good quality mRNA can be extracted for microarray analysis [7]. However, when performing such studies one has to be aware of the possibility that the differential expression could be caused by years of medicine usage or by pre- or post-mortem artifacts.

### Animal Models

The use of animal models for the analysis of complex neurodevelopmental disorders appears to be an attractive alternative to circumvent the problems encountered when human material is used for mRNA or protein profiling. However, animals do not exhibit higher-order functions, some of which may be associated with complex human disorders. Nevertheless, one can take advantage of specific characteristics (endophenotypes) of an animal model to study the genetic and environmental factors that lead to a particular phenotypic outcome.

Several categories of animal models may be employed, including models based on a behavioral selection (e.g. the endophenotype prepulse inhibition), on a pharmacological selection (e.g. the psychotic effects of drugs, such as amphetamine) or on brain lesions (e.g. animal models with disconnections of the hippocampus). In addition, genetic animal models – with targeted genetic manipulations of specific genes - can be used, including knockout and transgenic models. Genetically modified animals can be subjected to a whole battery of behavioral tests to understand the role of a gene in neurodevelopmental aetiology. Furthermore, such models can be used to study environmental manipulations, including maternal or chronic stress paradigms.

## SUSCEPTIBILITY LOCI AND GENES OF COMPLEX NEURODEVELOPMENTAL DISORDERS 

In the past decades, an impressive amount of linkage and association studies have been performed. However, conclusive evidence from the numerous genetic linkage and association studies has not yet been obtained. The studies have continuously led to inconsistent and controversial findings. In the next paragraphs, the genetic aetiology of the complex neurodevelopmental disorders is summarized. However, because positive associations are often published more easily one has to realize publication bias and the fact that even results obtained by meta-analysis may represent false positives.

### Autism

Autism has a prevalence of ~0.6% in the general population and is four times more prevalent in boys than in girls. Together with four other disorders (Asperger’s disorder, childhood disintegrative disorder, Rett syndrome and Personality Disorder Not Otherwise Specified) it belongs to the group of Pervasive Developmental Disorders (PDD). Autism is the most common PDD and usually appears during the first three years of life. Its symptoms include impairments in verbal and nonverbal communication, lack of social interaction, and restricted and stereotypical behaviour [8]. Though autism is one of the most hereditary disorders in psychiatry, with an estimated heritability of up to 90% (Table **2**) [9], the search for susceptibility genes has proven to be complex. Until now, a number of chromosomal loci have been identified that may represent regions predisposing to autism, including regions on chromosome 1p12-p21.1, 1q21-q44, 2q24.1-q33.1, 3q21.3-q29, 4q21.3-q35.1, 5p12-p15.33, 6q14.3-q23.2, 7q21.2q36.2, 10p12.1-p14, 10q23.3-q26.3, 13q12.13-q33.1, 15q13.1-q26.1, 16p12.1-p13.3, 17q11.1-q21.2, 19p13.11-p13.3 and 19q12-q13.12 [reviewed in 10]. Although most susceptibility regions have been studied in more detail *via *the candidate-gene approach (e.g. the Reelin gene on chromosome 7q22 and the serotonin transporter gene (SLC6A4) on chromosome 17q11.1-q12), no gene has been found to clearly contribute to autism susceptibility. Recently, the first GWA studies for autism have been reported with significant associations, including CNVs, found in several genetic loci [11-13], but the results have been inconclusive. Thus, despite the high heritability estimates for autism, its genetic aetiology still needs to be elucidated.

### Schizophrenia 

Schizophrenia is a common mental disorder affecting approximately 1% of the population [14]. It generally emerges between 16 and 30 years of age and is characterized by three main symptoms: cognitive disturbances, psychosis and negative symptoms [15]. Unfortunately, there are no genetic markers available for diagnosing schizophrenia. Therefore, diagnosis can only be based on clinical symptoms using the Diagnostic and Statistical manual for mental disorders version IV (DSM-IV, 2000) or the International Classification of Disease version 10 (ICD-10, 1992).

The first genetic studies on schizophrenia date back from 1916 and addressed the question whether the disorder has a genetic aetiology. Many subsequent family, twin and adoption studies clearly revealed the importance of a genetic component in schizophrenia [16] with an estimated heritability of around 80% (Table **2**) [17], but the responsible gene (or genes) is still elusive. Although many susceptibility loci have been identified, numerous inconsistent and controversial findings have been reported. The genes most often reported to be related to schizophrenia are the genes encoding disrupted in schizophrenia 1 (DISC1; 1q42.1), neuregulin-1 (NRG1; 8p12), dysbindin (DTNBP1; 6p22.3), D-amino acid oxidase activator (DAOA or G72; 13q34), D-amino-acid oxidase (DAO; 12q24), regulator of G-protein signaling 4 (RGS4; 1q23.3) and the dopamine-catabolizing enzyme catechol-O-methyl transferase (COMT; 22q11.21) [reviewed in reviewed in 18, 19]. However, relative risk effects of the variations range between 1.5 to 2.0, indicating only small-effect sizes. Recently, the first GWA study for schizophrenia using the Affymetrix GeneChip 500K Mapping Array Set on 178 schizophrenic patients and 144 controls has been reported. One SNP (rs4129148) close to the colony stimulating factor 2 receptor alpha chain gene (CSF2RA) on chromosome Xp22.32 and Yp11.3 showed association beyond the genome-wide significance threshold [20]. Independent replications to confirm this finding are however necessary. (For a more detailed overview of the genes reported to be associated with schizophrenia, we refer to http://www.polygenicpathways.co.uk/schizgenesandfunc.html).

### Bipolar Disorder

Bipolar disorder, also known as manic-depressive illness, is a severe mental disorder characterized by recurrent manic and depressive episodes causing dramatic mood swings. The prevalence of bipolar disorder is estimated to be 0.8-2.6% [21]. Although some have their first symptoms in childhood, most patients develop episodes in late adolescence or early adulthood. Bipolar disorder patients show many clinical features that are similar to those of schizophrenic patients, such as age of onset, psychotic symptoms, episodic courses of illness and a lifelong endurance. However, also clear distinctions exist between the two disorders. For example, bipolar disorder manifests as an impairment of mood, whereas schizophrenia is a primary disorder of cognition. Furthermore, most bipolar patients benefit from lithium therapy, whereas schizophrenics seldom do.

Twin and family studies have shown that bipolar disorder tends to run in families with an estimated genetic hereditary of 63% [22]. Interestingly, besides the shared clinical symptoms, bipolar disorder and schizophrenia may also share a genetic background [23]. A number of promising susceptibility genes for schizophrenia have been reported to associate with bipolar disorder as well, including G72, DAO, DISC1, NRG1, RGS4, COMT, neural cell adhesion molecule 1 (NCAM1; 11q23.1), brain-derived neurotrophic factor (BDNF; 11p13) glutamate receptor, metabotropic 3 and 4 (GRM3; 7q21.1-q21.2 and GRM4; 6p21.3), glutamate receptor, ionotropic, N-methyl D-aspartate 2B (GRIN2B; 12p12), megalencephalic leukoencephalopathy with subcortical cysts 1 (MLC1; 22q13.33), synaptogyrin 1 (SYNGR1; 22q13.1) and solute carrier family 12 (potassium/chloride transporters), member 6 (SLC12A6; 15q13-q15) [reviewed in reviewed in 21, 24]. Recently, The Wellcome Trust Case Control Consortium reported a GWA study on bipolar disorder using the Affymetrix GeneChip 500K Mapping Array Set and found one chromosomal region with strong evidence of association (16p12) and 13 regions with moderate association (2p25, 2q12, 2q14, 2q37, 3p23, 3q27, 6p21, 8p12, 9q32, 14q22, 14q32, 16q12 and 20p13) [25]. (For a complete list of the genetic associations with bipolar disorder, we refer to http://www.polygenicpathways.co.uk/Bipolargenes.html).

### Major Depression

Like bipolar disorder, depression is a major mood disorder. Although many clinical aspects are comparable between major depression and bipolar disorder, a number of characteristics are different between the two disorders: depression is much more heterogeneous, has a higher environmental contribution and has a higher prevalence with an overall lifetime risk of 16.2% in the United States [26]. Since the genetic contribution to major depression is only ~37% [27] and the illness is highly heterogeneous, unravelling its genetic pathogenesis is extremely difficult. To date, no clear genetic risk factors for major depression have been identified. Most studies have focused on well-known polymorphisms that have been hypothesized to associate with other psychiatric disorders. For example, the Val66Met variant in the BDNF gene, the short allele of the SLC6A4 gene, and the Val158Met variation in the COMT gene have been studied in depression cohorts [28-30], but the results are contradictory [31-33].

### ADHD

ADHD was first described in 1845 and affects up to 1 in 20 children [34, 35]. The principal problem for children with ADHD is the impairment to control their behaviour, due to inattention, hyperactivity and impulsivity. According to the DSM-IV guidelines, these symptoms should appear early in a child’s life, before age 7, and should continue for at least 6 months, otherwise the diagnosis ADHD is not justified. Other disorders often accompany ADHD, including learning disabilities, oppositional defiant disorder, conduct disorder, Tourette’s syndrome and/or depressive illness.

Twin studies have indicated a relatively high genetic contribution reaching an average of 70% [36]. Thus far, many candidate gene studies on ADHD have focused on the dopamine and serotonin pathways. Meta-analyses of the available data have suggested several of the genes belonging to either pathway to be involved in ADHD pathogenesis, including the dopamine receptors D4 (DRD4; 11p15.5) and D5 (DRD5; 4p16.1), SLC6A4, the dopamine transporter (DAT or SLC6A3; 5p15.3), the 5-hydroxytryptamine (serotonin) receptor 1B (HTR1B; 6q13), dopamine beta-hydroxylase (DBH; 9q34) and synaptosomal-associated protein of 25kDa (SNAP25; 20p12-p11.2) [reviewed in 36]. In addition, recently a large candidate gene analysis was performed involving 1,038 SNPs and spanning 51 candidate genes (belonging to the circadian rhythm genes and the dopamine, norepinephrine or serotonin pathways) that confirmed association with DRD4 and DAT1 [37], the two most replicated associations. 

### Tourette’s Syndrome 

Tourette’s syndrome (also called Gilles de la Tourette syndrome) is a neuropsychiatric disorder that occurs with an estimated prevalence of 1% among school-age children [38], is characterized by multiple chronic tics (involuntary movements and vocalizations) and is often accompanied by other behavioural disorders, including ADHD and obsessive-compulsive disorder (OCD) [34]. Although family and twin studies have suggested a contribution of genetic factors in Tourette’s syndrome, its precise contribution rate remains unclear [39]. Until now, most association studies have focussed on candidate genes belonging to the dopaminergic pathway, and showed several positive associations for the DAT, monoamine oxidase A (MAOA; Xp11.3) and the dopamine receptors D2 (DRD2; 11q23), D3 (DRD3; 3q13.3) and D4 [40-47]. However, since not all subsequent replication studies were positive [48-53] and other genes were found to associate as well [54], the contribution of the dopaminergic pathway to Tourette’s syndrome remains to be established. 

### Dyslexia (Reading Disability)

Dyslexia affects 5–10% of school-age children [55] and is characterized by problems with word recognition and spelling. Linkage studies have revealed a number of chromosomal susceptibility loci for dyslexia (1p34-p36, 2p16-p15, 3p12-q12, 6p21, 6q13-q16, 11p15, 15q21, 18p11 and Xq27) [reviewed in 56]. Within and near these loci several genes have been studied using association analyses, resulting in a few candidate genes for dyslexia: dyslexia susceptibility 1 candidate 1 (DYX1C1; 15q21) [57], roundabout Drosophila homolog 1 (ROBO1; 3p12) [58] and doublecortin domain-containing protein 2 (DCDC2; 6p22.1) [59], but again the results are not conclusive and therefore the genetic aetiology of dyslexia is currently still unclear.

### Epilepsy (Seizure Disorder)

Epilepsy is a heterogeneous group of disorders with abnormal electrical brain activity. In adults, temporal lobe epilepsy (TLE) is the most common form of epilepsy with an age of onset in late childhood or adolescence. In childhood, the most common form of epilepsy is febrile seizures (FSs), with a prevalence of 2-5% in Western countries and an estimated heritability of 70% [60]. Genetic linkage analyses have identified a number of loci for familial FS, including the loci on chromosome 19p13.3, 2q23-q24, 5q14-q15 and 18p11.2 containing the genes encoding casein kinase I gamma 2 isoform (CSNK1G2; 19p13.3), sodium channel, voltage-gated, type I, alpha subunit (SCN1A; 2q24.3), G protein-coupled receptor 98 (GPR98; 5q13) and inositol(myo)-1(or 4)-monophosphatase 2 (IMPA2; 18p11.2), respectively [61-64]. Although linkage of these loci has been replicated in some other familial cases, only for CSNK1G2 and IMPA2 association was found in subsequent association analysis [64, 65]. In addition, a number of other candidate genes have been identified *via *association studies, including the genes encoding cholinergic receptor nicotinic alpha 4 (CHRNA4; 20q13.2-q13.3), gamma-aminobutyric acid A receptor gamma 2 (GABRG2; 5q31.1-q33.1) and -beta 3 (GABRB3; 15q11.2-q12), interleukin 1 beta (IL1B; 2q14) and interleukin 1 receptor antagonist (IL1RN; 2q14.2) [66-70]. However, the latter associations could not be replicated in subsequent cohorts [71-75].

### Mental Retardation

Mental retardation (MR) occurs in approximately 2-3% of the population in developed countries [76]. For the diagnosis MR a number of criteria have to be fulfilled, including an IQ lower than 70 and behavioural disabilities that are already evident in childhood. The underlying causes of MR can be diverse, varying from inborn causes such as Down’s syndrome, Fragile X syndrome and fetal alcohol syndrome (these three causes are responsible for 30% of the MR cases [77]), but also malnutrition and problems during pregnancy or birth can increase the risk for MR [78].

Although it is evident that a genetic factor is involved in the aetiology of MR and the genetic cause of a number of subtypes has been identified (e.g. trisomy of chromosome 21 in Down’s syndrome), the majority of cases have an unknown genetic aetiology. Since MR has a clearly X-linked inheritance pattern and is more often found in males than females, variations in the X-chromosome may increase the risk for MR. A number of X-linked genes have been identified as susceptibility genes for MR, including fragile X mental retardation 2 (FMR2; Xq28), oligophrenin 1 (OPHN1; Xq12), p21 (CDKN1A)-activated kinase 3 (PAK3; Xq22.3-23), GDP dissociation inhibitor 1 (GDI1; Xq28), Rac/Cdc42 guanine nucleotide exchange factor (GEF) 6 (ARHGEF6; Xq26), ribosomal protein S6 kinase, 90kDa, polypeptide 3 (RPS6KA3; Xp22.2-p22.1), interleukin 1 receptor accessory protein-like 1 (IL1RAPL1; Xp22.1-p21.3), tetraspanin 7 (TSPAN7; Xp11.4), methyl CpG binding protein 2 (MECP2; Xq28), acyl-CoA synthetase long-chain family member 4 (ACSL4; Xq22.3-q23) and aristaless related homeobox (ARX; Xp21) [79-89]. However, many other genes are likely linked to MR.

### Addictive Disorders

Many twin studies have been performed on addictive disorders (both alcohol and drug abuse), which indicated heritability levels of 50-60% in alcohol consumption [90] and up to 70% in severe smoking [91]. Since the dopaminergic pathway plays a central role in the reward system, the genes involved in this pathway are thought to be susceptibility genes for addictive disorders. Indeed, a number of studies have identified polymorphisms in this pathway that infer susceptibility to addiction: genetic variations in the DRD2, DRD3, COMT and DAT1 genes have been reported to associate with smoking, alcoholism, cocaine abuse and heroin addiction [92-101]. Nevertheless, despite the large number of studies reporting association, meta-analyses have shown that the effects are only weak or not significant [102, 103].

### Fetal Alcohol Syndrome

During pregnancy, alcohol use by the mother may lead to fetal alcohol syndrome (FAS) that occurs at a rate of 0.5-2 individuals per 1000 live births. A number of family, twin and animal studies have suggested a genetic component in FAS pathogenesis, one of the main candidate genes being the alcohol dehydrogenase 1B (ADH1B) gene located on chromosome 4q21-q23. However, whereas some studies report a protective effect for a number of ADH1B subtypes, others were not successful in reproducing these results [reviewed in 104]. Besides ADH1B, other candidate genes have been suggested as risk factors for FAS pathogenesis, such as the cytochrome P450 2E1 gene (CYP2E1; 10q24.3-qter) [105, 106]. 

### Anxiety Disorders

Panic disorder, OCD, separation anxiety, overanxious disorder, agoraphobia and other phobias all belong to the group of anxiety disorders and are relatively common (lifetime prevalence of 25% [107]). Twin studies have indicated that a genetic factor is involved in anxiety disorders, but the genetic contribution to the disorders is only modest (30-40%) [108]. Yet, many linkage and association studies have been performed to determine the chromosomal locations or genes involved in the pathogenesis of the various subtypes of anxiety disorders. Panic disorder showed significant linkage to chromosomal regions 9q31, 13q and 22q [109, 110], for OCD linkage was reported to chromosome 1q, 3q27-28, 6q, 7p, 9p24, 10p15, 14 and 15q [111-114], and for other anxiety disorders linkage was observed for chromosome 14p (simple phobia) [115], 16 (social phobia) [116], 1q, 4q, 7p, 12q and 13q (neuroticism) [117] and 8p21-23 (harm avoidance) [118]. Recently, also genome-wide linkage analyses on individuals with a broad anxiety phenotype rather than based on the DSM-IV anxiety disorder diagnosis have been performed and significant linkage was observed for chromosome 14 [119] and 4q31-q34 [120]. 

Besides linkage analysis, many case-control design studies on candidate genes for anxiety pathogenesis have been performed. For panic disorder, a positive association was found for the serotonin receptors HTR1A (5q11.2-q13) and HTR2A (13q14-q21) [121, 122], COMT [123], the neuropeptide cholecystokinin (CCK; 3p22-p21.3) [124], the adenosine A2a receptor (ADORA2A; 22q11.23) [125], MAOA [126], the nuclear transcription factor cAMP-responsive element modulator (CREM; 10p11.21) [127], the peripheral benzodiazepine receptor (PBR or TSPO; 22q13.31) [128], glutamic acid decarboxylase 1 (GAD1; 2q31) [129], diazepam binding inhibitor (DBI; 2q12-q21) [130], calmodulin-dependent protein kinase kinase b (CaMKKb; 12q24.2) [131] and angiotensin-converting enzyme (ACE; 17q23.3) [132]. In addition, an association analysis of 90 SNPs located in 21 candidate genes revealed eight SNPs to be associated with panic disorder (located in the CCK, serotonin and dopamine systems), but all with a minor individual effect [133]. 

Besides association with panic disorder, a number of susceptibility genes have been found to associate with other subtypes within anxiety disorders as well, such as the serotonin system in OCD and neuroticism [134-139], MAOA in generalized anxiety disorder and neuroticism [140, 141], COMT in neuroticism and phobic anxiety [141, 142] and BDNF in anxiety-related personality traits [143, 144]. 

### Posttraumatic Stress Disorder 

Posttraumatic stress disorder (PTSD) can occur in a subset of individuals exposed to extreme traumatic events [145], and has a lifetime incidence of ~9–15% [146, 147], and an estimated genetic inheritance of ~30% [148]. Susceptibility genes for PTSD have not yet been identified, but to date the number of individuals screened is low, while the few genetic studies that have been performed mainly focussed on key candidate genes, including BDNF, neuropeptide Y (NPY; 7p15.1), the glucocorticoid receptor (NR3C1; 5q31.3), and components of the serotonin and dopamine pathways [149-153].

### Eating Disorders

Anorexia and bulimia nervosa are two major eating disorders with still unknown risk factors. For a long time, eating disorders have been considered to be caused by sociocultural factors. However, it has recently become clear that also genetics may play a substantial role in its aetiology. Family and twin studies have shown that heritability estimates for eating disorders vary from 48% to 74% in anorexia nervosa and from 55% to 83% in bulimia nervosa [154-157]. Since serotonin plays an important role in mood and feeding, genetic variations in the serotonergic pathway are thought to lead to eating disturbances. Indeed, a number of positive associations with the serotonin receptors HTR2A and HTR2C (Xq24), and also with the serotonin transporter gene have been reported [158-160], however, replication was not always successful [161, 162]. Furthermore, associations were found for BDNF [163, 164], the growth hormone secretagogue receptor (ghrelin receptor or GHSR; 3q26.31) [165] and COMT [166, 167]. 

### Spina Bifida

Spina bifida is caused by unsuccessful closure of the neural tube during early development (between embryonic day 17 and 30) and occurs with a frequency of 1-2 cases per 1000 births. The exact aetiology of spina bifida is poorly understood, but it is clear that both genetic and environmental factors are involved [168]. Since individuals with spina bifida often die prenatal or early postnatal and thus hardly any families exist with several affected members, this disease could well be the most difficult complex disorder to study at the genetic level. Based on animal and epidemiological studies, genes involved in folic acid (folate), vitamin B12 and homocysteine metabolism, or genes involved in neurulation have been hypothesized to play a role in spina bifida genesis [reviewed in 169]*.* However, until now, only a few genes have been reported to represent risk factors for spina bifida, including 5,10-methylenetetrahydrofolate reductase (MTHFR; 1p36.3) [170], methionine synthase reductase (MTRR; 5p15.3-p15.2) [171], platelet-derived growth factor receptor alpha (PDGFRA; 4q11-q13) [172] endothelial nitric oxide synthase 3 (NOS3; 7q36) [173] protein-L-isoaspartate (D-aspartate) O-methyltransferase (PCMT1; 6q24-q25) [174] and cofilin 1 (non-muscle) (CFL1; 11q13) [175]. 

### Hydrocephalus

Hydrocephalus occurs at a frequency of approximately 0.5 in 1000 births [176, 177] and is characterized by abnormal flow or resorption of cerebrospinal fluid. It is considered a heterogeneous complex disorder [178] with genetic and environmental aetiologies [179, 180]. Although approximately 37% of the hydrocephalus cases have a possible genetic aetiology [180], clear susceptibility genes for hydrocephalus have not been identified yet. Studies in animal models have suggested several loci as susceptibility regions for hydrocephalus, but these regions have not yet been reported as susceptibility regions in human [reviewed in 181]. 

## COMPLEX NEURODEVELOPMENTAL DISORDERS AND THE ENVIRONMENT

Since in general complex neurodevelopmental disorders have an estimated heritability lower than 100% (Table **2**), their aetiology includes another component that is thought to be primarily the environment (e.g. stressful life events). Numerous factors acting during early development of a foetus may contribute to the genesis of a neurodevelopmental disorder, including insufficient maternal nutrition, daily smoking, viral infection and repeated psychological stress [182]. Most environmental vulnerability factors are however difficult to assign and quantify.

The type and timing of the early environmental risk factors to which an organism is exposed appear to determine the phenotypic outcome. For example, a prenatal exposure of 9-days-pregnant mice to a sublethal intranasal administration of influenza virus led to both short-term and long-lasting deleterious effects on the developing brain structures and to abnormal behavior in the offspring of the mice [183]. Besides risk factors during early (prenatal) development, also obstetrical complications, including the use of resuscitation or an incubator, premature membrane rupture, diabetes, rhesus incompatibility, bleeding, preterm birth or caesarean birth, may increase the vulnerability to neurodevelopmental disorders [184, 185].

One obvious gene-environment link concerns the season in which birth took place. An excess of winter-spring births in bipolar disorder and schizophrenia has been observed [186]. A similar tendency has been found in schizoaffective disorder (December-March), major depression (March-May) and autism (March) [reviewed in 187]. Besides the season of birth, also the place of birth is thought to be associated. Urban–born (and brought-up) subjects are more susceptible to neurodevelopmental disorders than rural-born (and brought-up) subjects [188]. Furthermore, risk factors like immigration and adoption may contribute to the development of psychiatric disorders [189, 190].

## GENE-ENVIRONMENT INTERACTIONS IN COMPLEX NEURODEVELOPMENTAL DISORDERS

One of the reasons that the genetic and environmental factors in complex neurodevelopmental disorders are difficult to define is the fact that the two factors may interact. However, such an interaction may be complex and act at various levels. For instance, genetic and environmental factors may have an additive effect, genetic factors may affect the influence of the environment on a phenotype or environmental factors may modulate the expression of genetic variants. 

An example of a gene-environment interaction concerns the influence of stressful life events on depressive individuals with a functional polymorphism in the promoter region of the serotonin transporter gene. Individuals with the short allele have been found to respond differently to stressful life events (e.g. childhood maltreatment) and as such are more vulnerable to develop depressive symptoms than individuals with the long allele [29]. A second example of gene-environment interaction is the valine/methionine polymorphism (SNP rs4680) in the COMT gene. Upon cannabis use, individuals carrying the valine allele have a higher chance to exhibit psychotic symptoms and to develop schizophreniform disorders when compared to individuals with two methionine alleles [191]. 

## COMPLEX NEURODEVELOPMENTAL DISORDERS AND EPIGENETICS

Epigenetics is defined as heritable changes in gene expression patterns that occur without changing the DNA sequence itself [192], and includes DNA methylation and posttranslational modifications of histone proteins. DNA methylation, i.e. a covalent binding of a methyl group to the 5-position of the cytosine ring within the sequence 5’-CG-3’ (CpG), can be tissue- and cell-type specific and is found in all vertebrates, and many invertebrates and plants. CpG clusters with a minimum of 200 base pairs, a CG percentage greater than 50% and an observed/expected CpG ratio greater than 0.6 are called CpG islands. These islands are often found in gene promoter regions and can protect single CpGs within a CpG island from DNA methylation. 

An apparent link between the methylation status and gene transcription levels has led to the speculation that alterations in the methylation pattern (epimutations) might contribute to altered gene expression. Such epimutations are thought to occur upon exposure to environmental risk factors, including early developmental stress. Since early embryos seem to be particularly sensitive to epimutations [193, 194], this factor should be considered for the aetiology of neurodevelopmental disorders. For instance, epigenetic alterations are responsible for a number of neurodevelopmental disorders with single-gene defects, such as Rett Syndrome, ICF Syndrome, Fragile X Syndrome and ATR-X Syndrome [195-198]. A role for DNA methylation has also been proposed in connection with complex neurodevelopmental disorders. For example, spina bifida can be caused by a lack of folate [reviewed in 199], a compound needed for the generation of S-adenosylmethionine (SAM) that donates the methyl group in the DNA methylation process. Also, some patients with depressive illness and schizophrenia display lower serum folate levels [200]. Animal models further provide evidence for a possible link between epigenetics and neurodevelopmental disorders. Following a diet with L-methionine, a precursor in the biosynthesis of SAM, the reeler mouse (a model for schizophrenia) showed increased promoter methylation of the reelin gene, reduced reelin expression and a declined prepulse inhibition of startle. These effects could subsequently be reversed by valproic acid, a mood-stabilizing drug used for treatment of epilepsy, bipolar disorder and schizophrenia [201]. In addition, the adult offspring of rat mothers that showed high licking and grooming (LG) and arched-back nursing (ABN) (two forms of maternal behaviour in the rat that serve as the basis for the individuals programming of the stress response) are less fearful, have a lower hypothalamic-pituitary-adrenal response to stress, and have a lower DNA methylation status in the promoter region of the glucocorticoid receptor gene when compared to the offspring of low-LG and -ABN mothers [202]. Thus, alterations in epigenetic profiles may contribute to the generation of complex neurodevelopmental disorders. 

## COMPLICATING FACTORS IN ASSOCIATION STUDIES

Although numerous studies suggest that genetic variants play a significant role in the aetiology of complex neurodevelopmental disorders, in almost all cases the precise genetic background remains to be identified. Several factors (see below) have greatly complicated the identification of the genetic basis of neurodevelopmental disorders.

### Definition of Phenotype

In the genetics of psychiatric disorders, the definition of a phenotype is one of the main problems. Most genetic studies use patient characterization according to the DSM-IV or ICD-10 criteria. However, investigators nowadays believe that the phenotype should be specified in more detail, since most neurodevelopmental disorders include a number of intermediate clinical subtypes and distinct phenotypical parameters (endophenotypes) [203], presumably each with a different genetic background. Such endophenotypes may help in the identification of risk factors, although the effectiveness of this approach has recently been questioned [204]. Nevertheless, analysis of an endophenotypically defined group of patients may increase replication efficacy.

### Population Stratification 

Genetic variations often occur among (geographically or ethnically) different populations and this fact may thus increase the difficulty in data interpretation as well. For correct stratification and successful replication, it is therefore highly important that samples are clinically, geographically and ethnically well characterized.

### Gene-Environment Interactions

Another complicating factor in the identification of susceptibility genes for neurodevelopmental disorders concerns the possibility of multifactorial gene-plus-environment interactions, as mentioned above. Unfortunately, such interactions are still difficult to quantify and interpret. 

### Multiple Genes Hypothesis

The search for susceptibility genes is further complicated when the aetiology of a complex neurodevelopmental disorder can not be explained by a single genetic variant with a relatively large effect but is rather caused by an interplay of a number of genes with small (additive) effects. For schizophrenia pathogenesis, the essential parameters for single- and multiple-locus models have been calculated and an interaction of about three different genes together with the environment was predicted to underly this disorder [205]. Not surprisingly, in general the greater the number of genes involved the more difficult their identification will be and larger (or additional) cohorts will be necessary to reach significant association. In addition, besides the possibility that multiple susceptibility genes are involved, individuals may be affected by the absence of protective alleles, while epistasis may also play a role.

### Common Disease-Common Variant or Rare-Variant Hypothesis

At present, it is not clear whether only a relatively small number of common genetic variants are linked to the aetiology of neurodevelopmental disorders (known as the “common disease-common variant hypothesis”, often abbreviated CD-CV) [206, 207], or if a large number of rare genetic variants is involved (“rare-variant-hypothesis” or heterogeneity hypothesis) [208]. In case of CD-CV, association analyses (e.g. GWA studies) may detect genetic variants if the studies contain enough power. It is clear that association analyses will be more difficult in rare variant cases [209].

## CONCLUSIONS AND FUTURE DIRECTIONS

Our summary of the current knowledge of the genetic, environmental and epigenetic contribution to the aetiology of neurodevelopmental disorders illustrates that unravelling the pathogenesis of these disorders is highly complex. Although further insights into the degree of the genetic contribution to the aetiology of neurodevelopmental disorders has been obtained, the identities of the genes involved and thus diagnostic markers are mostly lacking. 

Up to now, in general, a presumptive susceptibility gene appears to be linked to a single neurodevelopmental disorder. However, the existence of a single susceptibility gene for both schizophrenia and bipolar disorder [reviewed in 21, 24] illustrates that we probably have to await the results of future genetic research for a definitive conclusion concerning this issue. The future results will also reveal which pathways are involved in the complex disorders.

For a better understanding of the aetiologies involved, it will be fruitful to obtain detailed clinical, ethnical and geographical information on large groups of individuals. In addition, the environmental factors need to be well defined and documented. However, until now our knowledge of the relevant environmental risk factors is rather limited. Clearly, close collaborations between psychiatrists and genetic researchers are required. 

Undoubtedly, in the near future many more GWA studies with the 500K and even larger SNP arrays will be reported. Such studies will however not cover all genetic variations in the genome [210], because SNPs with a low minor allele frequency (MAF) (<0.05) are usually not included on the arrays, thereby excluding analysis of rare genetic variants. This is unfortunate, since the rare genetic variants are generally considered to have a higher chance of being causative [211]. Thus, candidate gene approaches of selected SNPs with a low prevalence may increase the chances to identify functional genetic variants. Because one would expect that causal SNPs have an effect in any population, a further consideration may involve a choice of SNPs with a low MAF in all ethnical populations. In this connection, one has to be aware of the possibility that such a SNP may need an additional polymorphism(s) to explain the phenotype (multiple genes hypothesis), while the additional genetic variation(s) may not be present in a particular ethnical population. One of the practical problems in dealing with low-MAF SNPs concerns the sample size necessary to obtain reliable association data, i.e. the lower the MAF the more samples are required to reach statistical significance. It is likely that inclusion of potentially functional SNPs (non-synonymous SNPs and SNPs in gene promoter regions or exon-intron boundaries) will increase the success rate in the analysis. At present, chips containing 20,000 non-synonymous human SNPs from ~11,000 genes are already available (www.affymetrix.com).

This review has attempted to provide an overview of the aetiologies of complex neurodevelopmental disorders. Clearly, many questions remain unanswered with respect to the pathogenesis of such disorders. Nevertheless, it is to be expected that within the next years the tsunami of genetic research will lead to more insights into the susceptibility genes. This new information can then be applied to start new research strategies, including the use of genetically manipulated cells or animal model systems carrying the susceptibility gene for functional studies on the pathways involved. Eventually, the acquired understanding of the molecular mechanisms underlying complex disorders may lead to translational research, including the design of gene/pathway-specific drugs and the application of disease-preventing strategies.

## Figures and Tables

**Table 1.** Neurodevelopmental Disorders and their Genetic Aetiologies

Group	Disorder	Genetic Aetiology
I (Aneuploidy)	Down’s syndrome	Trisomy of chromosome 21 (OMIM #190685).
II (Micro-deletion)	Prader-Willi syndrome / Angelman syndrome	~4 Mb deletion (~7 genes) of chromosome 15q11-q13 (OMIM #176270 and #105830).
	Smith-Magenis syndrome	Deletion (3.7 Mb) of chromosome 17p11.2 (OMIM #182290).
	DiGeorge/velo-cardio-facial syndrome	Hemizygous deletion (1.5 to 3.0-Mb) of chromosome 22q11.2 (OMIM #188400 and #192430).
	William’s-Beuren syndrome	Deletion of chromosomal region 7q11.2 (OMIM #194050).
III (Single-gene defect)	ATR-X syndrome	Mutations in the ATR-X gene on the X-chromosome (OMIM #301040)
	Barth syndrome (X-linked cardioskeletal myopathy and neutropenia)	Mitochondrial functional impairments due to the tafazzin (TAZ) gene on chromosome Xq28 (OMIM #302060).
	Fragile-X syndrome	CCG repeat expansion of the FMR1 gene (OMIM #300624).
	ICF syndrome	Mutations in the DNA methyltransferase 3B (DNMT3B) gene on chromosome 20 (OMIM #242860).
	Neurofibromatosis	Mutations or deletion (~1.5 Mb) in the neurofibromin gene on chromosome 17q11.2 (OMIM +162200).
	Rett syndrome	Mutations in the MeCP2 gene on the X-chromosome (OMIM #312750).
	Smith-Lemli-Opitz syndrome	Mutations in the gene encoding sterol delta-7-reductase (DHCR7) on chromosome 11q12-q13 (OMIM #270400).
IV (Multifactorial)	Addictive disorders	Multiple genes (?)
	Attention deficit (hyperactivity) disorders	
	Anxiety disorders	
	Asperger’s disorder	
	Autistic disorders	
	Depressive illness	
	Dyslexia (reading disability)	
	Eating disorders	
	Epilepsy (seizure disorder)	
	Fetal alcohol syndrome	
	Hydrocephalus	
	Manic depressive illness (bipolar disorder)	
	Mental retardation	
	Schizophrenia	
	Spina bifida	
	Tourette’s syndrome	

**Table 2.** Estimated Heritability of Complex Neurodevelopmental Disorders

Disorder	Estimated Heritability	References
Autism	>90%	[9]
Schizophrenia	80%	[17]
ADHD	70%	[36]
Epilepsy	70%	[60]
Drug addiction	70%	[91]
Spina bifida	70%	[212]
Bipolar disorder	63%	[22]
Eating disorders	48-74%	[154, 156]
Dyslexia	50-70%	[213]
Alcohol addiction	50-60%	[90]
Panic disorder	30-46%	[214]
Posttraumatic stress disorder	30%	[148]
Obsessive-compulsive disorder	26-47%	[215, 216]
Anxiety disorders	30-40%	[108]
Depressive illness	37%	[27]

## ABBREVIATIONS

ABN: = Arched-back nursing
ADH1B: = Alcohol dehydrogenase 1B
ADHD: = Attention deficit hyperactivity disorder
ATR-X: = X-linked alpha thalassemia/mental retardation
BDNF: = Brain-derived neurotrophic factor
CCK: = Cholecystokinin
CD-CV: = Common disease-common variant
CGH: = Comparative genome hybridization
CNV: = Copy-number variants
COMT: = Catechol-O-methyl transferase
CSNK1G2: = Casein kinase I gamma 2 isoform
DAO: = D-amino-acid oxidase
DAOA: = D-amino acid oxidase activator
DAT: = Dopamine transporter
DISC1: = Disrupted in schizophrenia 1
DRD2: = Dopamine receptor D2
DRD3: = Dopamine receptor D3
DRD4: = Dopamine receptor D4
DSM: = Diagnostic and Statistical manual for mental disorders
FAS: = Fetal alcohol syndrome
FMR1: = Fragile X mental retardation 1
FS: = Febrile seizures
GWA: = Genome-wide association
HTR2A: = 5-hydroxytryptamine (serotonin) receptor 2A
ICD: = International Classification of Disease
ICF: = Immunodeficiency, centromeric region instability, facial anomalies
IMPA2: = Inositol(myo)-1(or 4)-monophosphatase 2
LG: = Licking and grooming
MAF: = Minor allele frequency
MAOA: = Monoamine oxidase A
MeCP2: = Methyl CpG binding protein 2
MR: = Mental retardation
NRG1: = Neuregulin-1
OCD: = Obsessive-compulsive disorder
PDD: = Pervasive developmental disorders
PTSD: = Posttraumatic stress disorder
RGS4: = Regulator of G-protein signalling 4
SAM: = S-adenosylmethionine
SLC6A4: = Serotonin transporter
SNP: = Single-nucleotide polymorphism
